# Numerical Investigation of the Fire Behavior of Storage Rack Systems Protected by Intumescent Coating

**DOI:** 10.1021/acsomega.2c05287

**Published:** 2022-09-28

**Authors:** Burak Kaan Cirpici, Suleyman Nazif Orhan, Casim Yazici, Fatih Mehmet Özkal

**Affiliations:** † Department of Civil Engineering, Erzurum Technical University, 25050 Erzurum, Turkey; ‡ Department of Construction, Ağrı İbrahim Çeçen University, 04400 Ağrı, Turkey; § Department of Civil Engineering, Atatürk University, 25240 Erzurum, Turkey

## Abstract

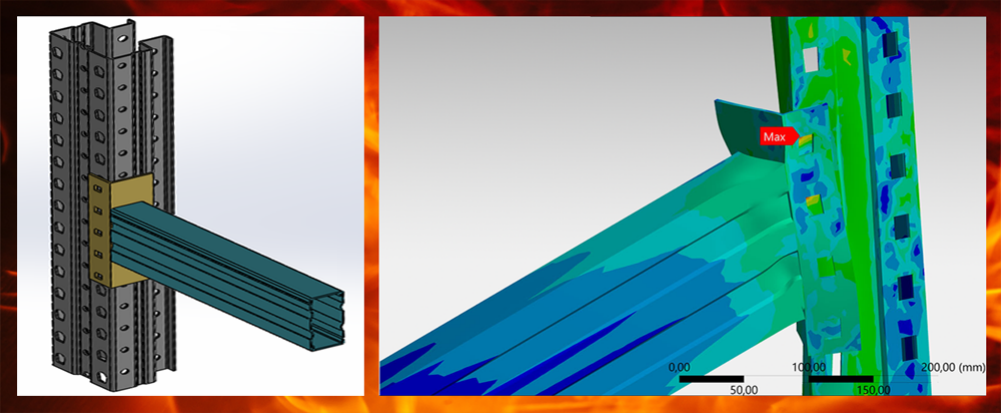

Using a finite element strategy, this study investigates the behavior of beam-to-column connections in storage rack systems exposed to high temperatures. The purpose of this research was to develop moment–rotation (*M*–θ) curves after painting various structural members with varied configurations in order to evaluate the performance of intumescent-coated beams, uprights, and connectors, which are components of storage rack systems. Within the scope of this work, finite element analyses were carried out in two stages. First, thermal analyses were performed using the transient thermal analysis system of ANSYS Workbench software to estimate the ultimate temperatures of the beam, upright, and connector, which were painted with 1 mm thick paint and exposed to standard (ISO-834) fire. The results were then compared to the Eurocode 3 Part 1.2 with a satisfactory agreement. In the second stage of the analysis, a total of 9 possible alternative models were investigated in the static structural analysis system, reflecting the effect of applying fire protection to the different portions of the rack system. Since the most critical stress level is achieved around the connector tabs, it has been observed that protection of the connector in individual or binary conditions provides higher performance while protection of the beam causes divergent joint behavior. Additionally, comparison of fully protected and unprotected conditions presents an increment of more than 7% on the joint’s ultimate moment capacity and initial stiffness, which is an explicit contribution of the intumescent coating to fire resistance.

## 1 Introduction

The necessity of product storage has risen dramatically in recent years as the distance sales and logistics sector has grown rapidly. To further understand the behavior of these structures, researchers used finite element analyses and experimental investigations. Storage rack systems are the 3D product of cold formed steel. Storage rack systems consist of uprights (columns), beams, connectors, and interconnection elements. Beam-to-column joints in storage rack systems connect the beam and column via a beam end connector. This connector allows the construction to be dismantled and reconstructed according to the storage needs and convenience of beam-to-column joining. In storage rack systems, commercial software could not be developed due to the variation of tab designs on the connector in beam-to-column connections.^1^ Design codes such as ANSI MH16.1,^2^ EN 15512,^3^ and AS 4084^4^ recommend that experimental investigations be used to predict the behavior of beam-to-column joints in storage rack systems. A study of the distortional buckling test method for steel storage rack columns is presented by Casafont et al.,^5^ proposing that the columns be subjected to linear buckling analysis to remedy the problem. With the use of newly created programs based on the finite strip approach and generalized beam theory, practical designers may now easily perform linear buckling analysis.

Fire is a serious threat to all building elements, resulting in loss of life and property. Storage rack systems are densely packed with products at substantial heights of up to 40 m, creating optimum conditions for fire spread. Cold-formed steel products have greater slenderness, less buckling resistance, and a higher thermal conductivity than regular steel. When cold formed parts are exposed to fire, the temperature rises quickly, reducing the strength of the materials employed in the structural members’ formation. This event leads to a rapid loss of strength and rigidity of the entire structure, resulting in premature and undesired failure of the structure. Alabi-Bello et al.^6^ discuss the findings of finite element simulations that led to the development of a design technique for transversely loaded thin-walled steel beams prone to local and distortional buckling failures at elevated temperatures using the direct strength method (DSM), which is a good approach for thin-walled steel members with nonuniform increased temperature distributions in the cross-section, according to the primary findings of this study. The results of a full-scale site fire test on a cold-formed steel portal frame building with semirigid joints are presented by Johnston et al.^7^ The goal of the research was to develop a performance-based approach for designing such structures under fire conditions. The research also showed that the semirigidity of the joints should be considered in the design. The single portal and entire building geometry models were nearly identical to the site test findings. Aliş et al.^8^ proposed an algorithm using material properties such as thermal conductivity, specific heat, and stress–strain relationship to capture the thermal and structural response of reinforced concrete beams under the Standard Fire Effect (ISO-834) numerically using ANSYS. It was discovered that the fire effect improved the load-bearing capacity of RC beams up to a certain temperature and then rapidly reduced it as the ambient temperature rose.

For more than 20 years, intumescent coatings have been utilized to safeguard structural steel in high-rise structures from fire-induced structural collapse. Intumescent coatings are becoming more popular in facilities that require strong fire protection, such as stadiums, skyscrapers, and multistory buildings, since they produce a high-quality finish and have good fire ratings. When exposed to fire, an intumescent coating can swell up to 100 times its original size (i.e., from 1 mm to 10 cm thick foam) in a regulated manner, forming a carbonaceous protective char. The char works as a heat transmission barrier, protecting the coated steel structure physically and thermally. In the event of a fire, the coatings can operate as passive fire protection for steel, which loses half of its load bearing capability at 500 °C, minimizing structural damage and extending evacuation time, preventing the loss of life and property.^9−15^ Organic and inorganic components are bonded together in a polymer matrix to form thermally reactive intumescent coatings.^16−18^ An acid source (“catalyst”), a carbonaceous chemical (“carbonific”), a blowing agent (“spumific”), binders, and additives are typically used in intumescent coating compositions; their functions have been extensively detailed in the literature, and formulas have been refined over the last decades to produce an effective protective char. The intumescent process of intumescent coatings can be separated into four steps: reaction, swelling, char formation, and char degradation.

1. Reaction (melting step): When an inorganic acid source is exposed to a heat source and reaches a certain temperature, the surface melts and converts into a viscous fluid.^19^
2. Swelling (expanding step): Endothermic reactions occur after the melting process, taking heat from the substrate and decomposing it, releasing a high number of gaseous products. The trapped gas bubbles cause the molten matrix to swell to 2–100 times its initial thickness, depending on the quality of the intumescent paint (coating), resulting in a porous medium with low density and thermal conductivity that serves as a thermal barrier for the metal substrate. The swelling process continues until either the blowing ingredient runs out or the carbon matrix becomes too viscous to keep the gas bubbles contained.
3. Char formation (charring step): With increasing temperature, the porous material hardens and releases the remaining volatiles, resulting in the formation of char. The char structure is strongly carbonaceous and has a black-gray color on the exposed surface at this stage.
4. Char degradation (change of char structure step): As the carbonaceous char oxidizes and CO_2_ is released, the black and compact char structure progressively transforms into a white, brittle, powdery foam at the exposed surface.

Fires in storage rack systems affect the building locally or completely with serious fire resistance losses in semirigid joints.^20^ Therefore, in recent studies, the behavior of semirigid joints between cold formed members under the influence of fire has been investigated.^1,21,22^

Design codes for storage rack systems do not provide any specific guidelines for estimating the moment–rotation–temperature (*M*–θ–*T*) relationships of beam-to-column connections. Based on the previous studies, it has been observed that fire retardant paints are not widely used in storage rack systems due to the high paint costs.

This study investigates the behavior of beam-to-column connections in storage rack systems exposed to high temperatures using the finite element method. The aim of this study is to evaluate the performance of intumescent-coated beams, columns, and connectors, which are components of storage rack systems, by generating moment–rotation (*M*–θ) curves after painting various structural members with varying configurations. To examine all the cases, it is desired to compare all of these three components unpainted, their binary combinations, and all coated cases among themselves. The samples were then used to evaluate the moment–rotation curves and ductility of beam-to-column joints in storage rack systems at elevated temperatures using the ISO-834 fire curve and the single cantilever test method. A nonlinear three-dimensional finite element (FE) model was developed to simulate high temperature experimental investigations on the commercially available software ANSYS, and the results were validated with the results in Eurocode 3 Part 1.8.^23^

## 2 Materials and Methods

### 2.1 Thermal Analyses

FEM analyses within the scope of the study herein were carried out in two stages. First, thermal analyses were carried out with the transient thermal analysis system in ANSYS Workbench software to determine the final temperatures of the beam, column, and connector, which were coated with 1 mm thick paint exposed to Standard (ISO-834) fire. The obtained results were compared with the Eurocode 3 Part 1.2.^24^ According to EN 1993-1-2, the temperature of a protected steel section is calculated using eq 1.
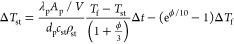
1with  where λ_p_ (W/(m K)) is the thermal conductivity of the fire protection material (intumescent coating), *A*_p_/*V* (m^–1^) is the section factor of the protected steel section, *d*_p_ (m) is the fire protection thickness, *c*_p_ (J/(kg K)) and ρ_p_ (kg/m^3^) are specific heat and density of the protection material, *T*_f_ (°C) and *T*_st_ (°C) are the exposed fire temperature (ISO-834) and steel temperature, respectively, and Δ*t* (s) is the time interval in seconds. The elastic modulus and yield strength values of the material according to the final temperatures determined in the sections were obtained by the study of Chen et al.^25^

Through the thermal analysis, final temperatures of the structural members were acquired under the influence of fire, and material properties of the steel were determined according to these temperature values. In the structural analysis, physical behavior of the loaded joint was investigated using modified material properties. Thus, the fire effect was reflected on the joint and the members through the alteration of mechanical characteristics.

### 2.2 Material Properties

#### 2.2.1 Thermal Properties of Steel

Type of the steel material is S355 for this study with yield strength of 355 MPa and tensile strength of 500 MPa. Eurocode 3 Part 1.2 was used to determine the thermal conductivity, specific heat, and density of structural steel.^24^ The structural steel utilized in the model has a density of 7850 kg/m^3^.

#### 2.2.2 Thermal Properties of Intumescent Coating

The influence of density and specific heat on the protected steel temperature in the validation investigation is minimal because the thickness is so modest. As a result, the constant values from Annex E of EN 13381-8:2013^26^ were used. The specific heat value is 1000 J/(kg·K), while the density value is 100 kg/m^3^. The viscosity of the fire-retardant coating is taken as 11000 mPa·s at 25 °C. However, the effective thermal conductivity has been obtained by Wang et al.^27^ based on their fire tests, and the furnace temperature followed the ISO-834. Fire has been applied to the protected surfaces in their model, which is also applicable in the mentioned study. Cirpici et al.^28,29^ proposed that if the fire condition is less severe than the Standard fire condition, the effective thermal conductivity of the coating retrieved from the Standard fire test can be utilized to estimate steel temperatures for other heating circumstances. In the model, the convective heat transfer coefficient is set to 25 W/(m^2^·K), and the emissivity of the coating for radiation is taken as 0.92.^24^ The intumescent coating expansion mechanism that leads to the ultimate char has not been studied in this study.

## 3 Results and Discussion

### 3.1 Comparison of Steel Temperatures (Eurocode-ANSYS Results)

**Figure 1:**
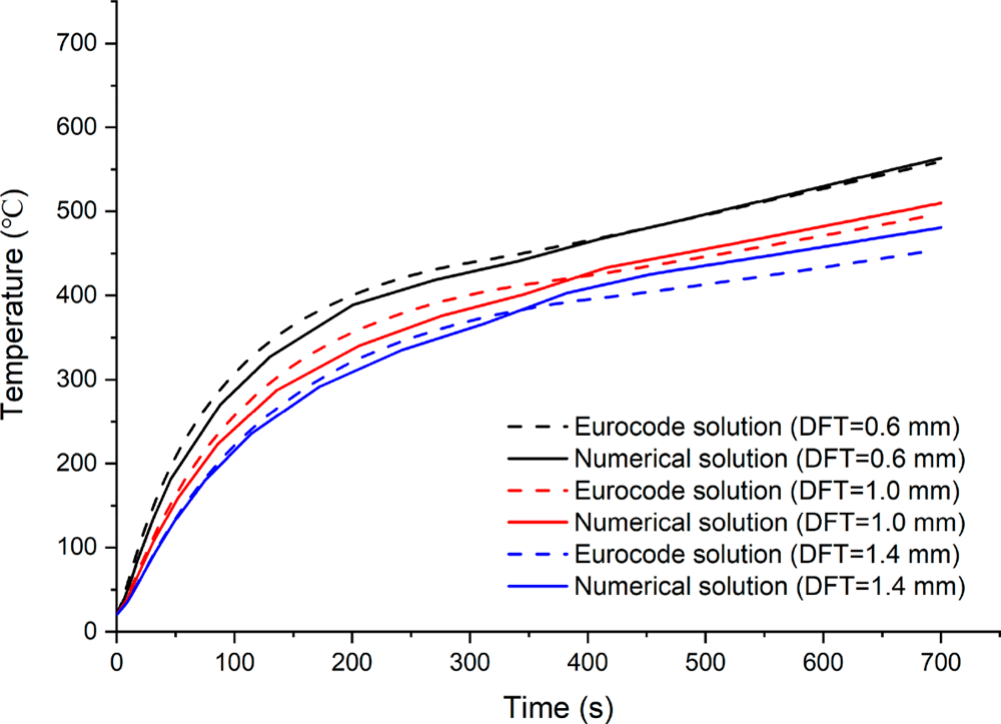
Comparison of beam temperatures with various intumescent coating thickness.

**Figure 2:**
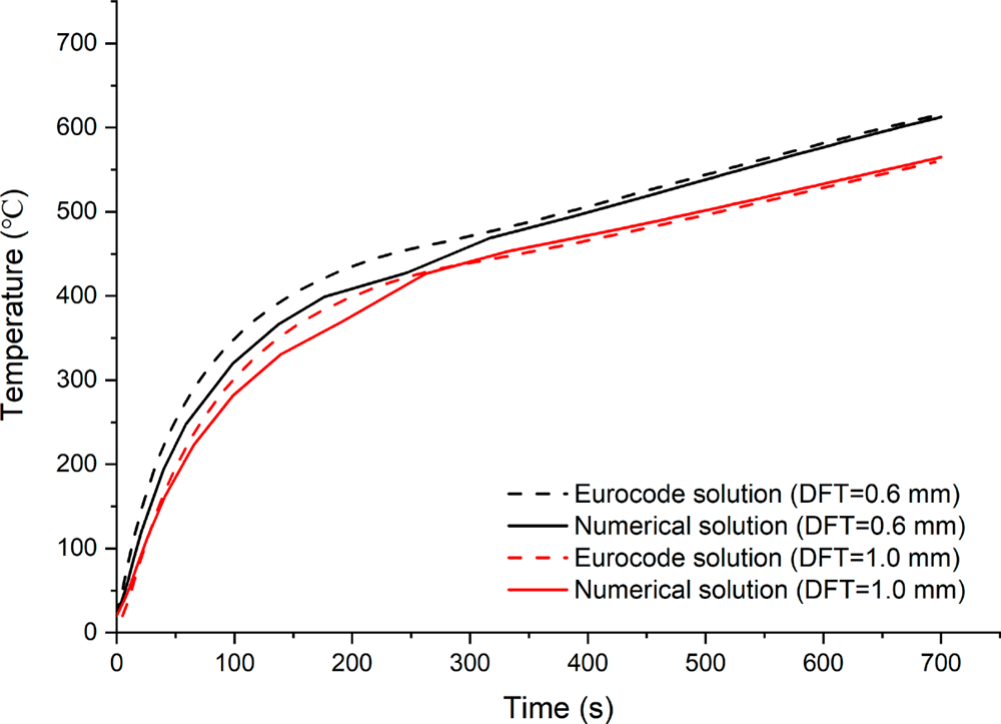
Comparison of upright temperatures with various intumescent coating thickness.

**Figure 3:**
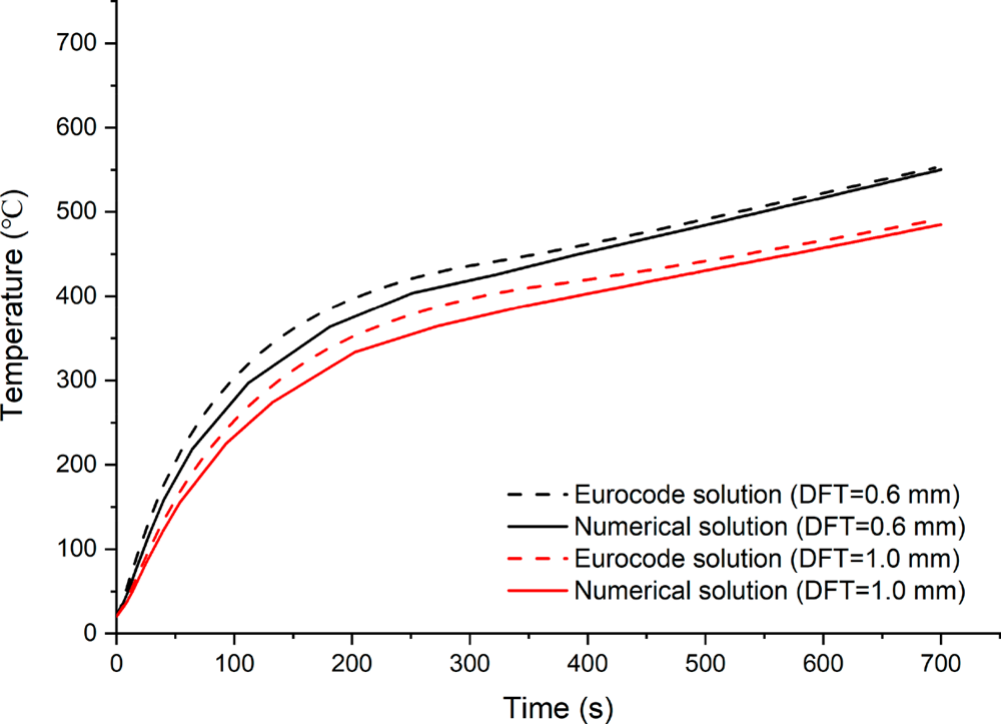
Comparison of connector temperatures with various intumescent coating thickness.

To demonstrate the numerical method’s confidence, a validation study comparing Eurocode solution and ANSYS results of structural members’ steel temperatures in terms of beam, upright, and connector was conducted. The beam temperature was determined using dry film thicknesses (DFT) of 0.6 mm, 1.0 mm, and 1.4 mm for the intumescent coating. For all coating thicknesses, a very satisfactory agreement was found, as illustrated in Figure 1. The results of 1.0 mm DFT thickness differ from those of 1.4 mm DFT thickness by a little margin. As a result, the 0.6 mm and 1.0 mm have been chosen for confirming upright and connection temperatures. Figures 2 and 3 show the results of the upright and connection comparisons, respectively. As can be seen, the numerical model’s validity is confirmed by the close findings.

### 3.2 Mechanical Properties of Steel at Elevated Temperature

**Figure 4:**
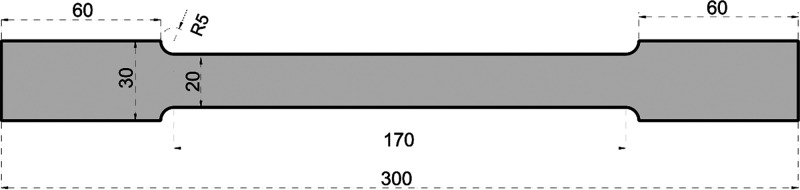
Tensile coupon test specimen (units in mm).

Beam, column, and connectors are manufactured from cold-formed steel. Mechanical properties of these materials were determined by tensile coupon tests according to ASTM E8 standards.^30^ The tensile test was carried out on a tensile testing machine with a capacity of 250 kN at a tensile speed of 25 mm/min. Dimensional properties of the tensile coupon test and mechanical properties are given in Figure 4 and Table 1, respectively. For coupon tests of cold-formed steel, samples with the cross-section properties of 1.5 mm, 2.5 mm, and 3 mm were prepared and kept at the target temperature of 700 °C for 20 min.^25^ After the samples were taken out of the oven, they were kept at room temperature. Table 2 presents the reduction factor verification of cold-formed steel at increasing temperature (700 °C) and decreasing temperature (20 °C).

**Table 1:** Material Properties of Test Specimens

				25 °C		700 °C	
member	Young’s modulus (GPa)	Poisson’s ratio	thickness (mm)	yield stress (MPa)	ultimate stress (MPa)	yield stress (MPa)	ultimate stress (MPa)
upright	210	0.3	2.5	430	569	370	424
beam	210	0.3	1.5	328	400	307	344
beam end connector	210	0.3	3.0	353	441	330	370

**Table 2:** Reduction Factor Verification of Cold-Formed Steel at Increasing and Decreasing Temperatures

		experiment		Chen et al.^25^	
maximum temp *a* (°C)	load testing temp *b* (°C)	*f*_*a*–*b*,*y*_/*f*_20–20,*y*_	*f*_*a*–*b*,*u*_/*f*_20–20,*u*_	*f*_*a*–*b*,*y*_/*f*_20–20,*y*_	*f*_*a*–*b*,*u*_/*f*_20–20,*u*_
700	20	0.935	0.839	0.882	0.862
700	20	0.936	0.860	0.882	0.862
700	20	0.860	0.745	0.882	0.862

**Figure 5:**
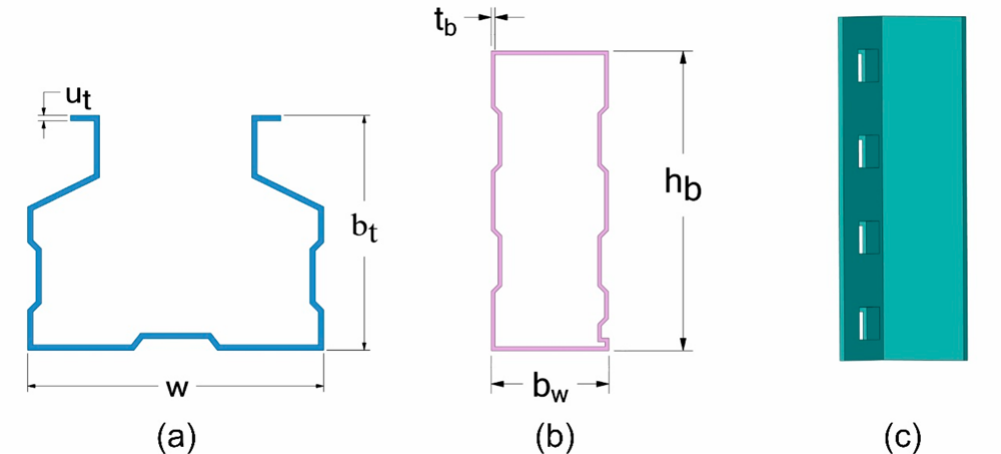
Details of joint members: (a) upright; (b) beam; (c) connector.

Individual postfire loading behavior of beams, columns, and connectors, which are structural components of storage rack systems, was examined using fire-retardant paint. Each of the components was painted one by one, and the consequences of beam–column–-connector fires were studied. After that, the fire effect was investigated as a binary combination. Finally, the fire impact of the entire system was evaluated, both with and without paint. Details of connection components for upright, beam, and connector have been presented in Figure 5. While the wall thickness (*u*_t_) of the column sections is 1.8 mm, the section width (*w*) is 100 mm, and the section height (*b*_t_) is 80 mm. The width of the beam sections (*b*_w_) is 50 mm, the section height (*h*_b_) is 120 mm, and the wall thickness of the section is 1.5 mm. The connector wall thickness is 3.5 mm with width of 39.5 mm, depth of 60 mm, and connector length of 240 mm.

### 3.3 Structural Analyses

In the second stage of the analysis, a total of 9 different alternative models were analyzed in the static structural analysis system, reflecting the effect of applying fire protection to different parts of the rack system under loading at postfire condition. In the analysis, a vertical displacement of 15 cm was applied to the beam from its middle part, and moment–rotation curves of the beams were obtained by considering the force value corresponding to this load and the application point of the displacement. In addition, the highest von Mises stresses in the system were determined and comparisons were performed. Boundary conditions and values stated below (in the validation study) were considered in the mesh application.

#### 3.3.1 Validation Results and Mesh Study

**Figure 6:**
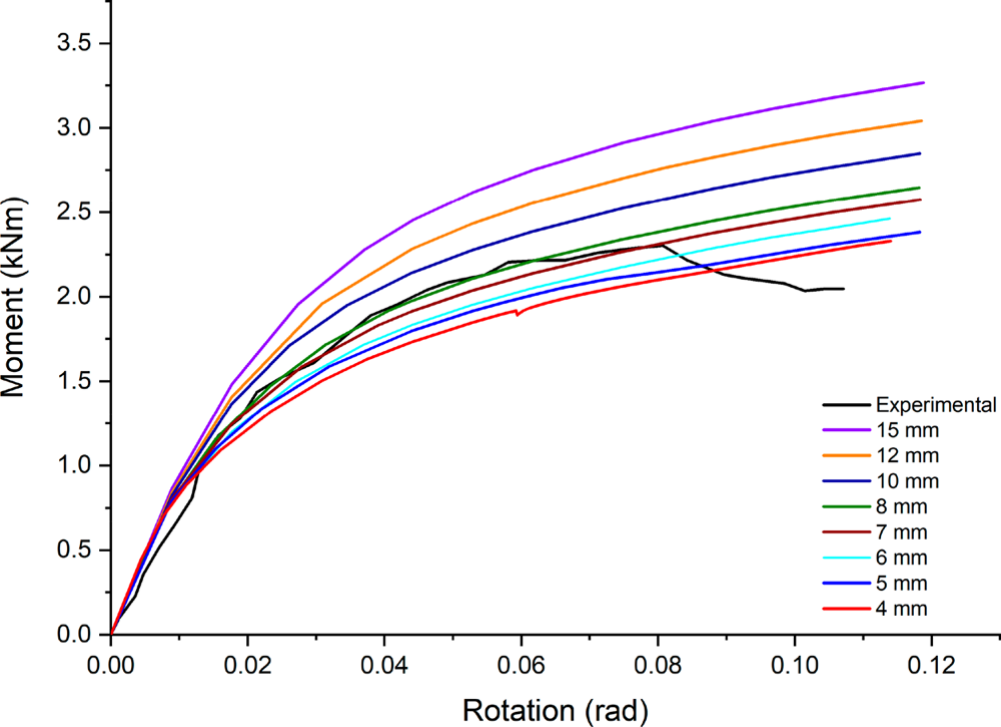
Moment–rotation results based on various element sizes.

To demonstrate the accuracy of the structural analyses made within the scope of the study, the analysis of the rack system, which was experimentally examined in the study carried out by Prabha et al.,^31^ was primarily carried out. In the analyses, the column was fixed from its upper and lower faces (fixed support), and a vertical displacement of 18 cm was applied to the area of 40 mm × 50 mm at the end of the beam. Displacement of the beam in horizontal directions was constrained in terms of compliance with the experimental conditions. The moment–rotation curves of the beams were obtained by considering the force value corresponding to the applied load and the application point of the displacement. Bonded contact was applied between beam and connector, while frictional contact was assumed between connector and column with a friction coefficient of 0.2.^32^ In the meshing of the elements, hexahedral elements were not preferred due to gaps and curvilinear parts, and tetrahedral (Tet10 – SOLID187) elements were used by applying the patch conforming method. SOLID187 element is well suited to modeling irregular meshes and defined by 10 nodes having three degrees of freedom at each node. This element has plasticity, hyperelasticity, creep, stress stiffening, large deflection, and large strain capabilities. In the validation study, a mesh convergence study was also performed, and the most appropriate mesh size was determined. Comparison of moment–rotation curves obtained using different element sizes is given in Figure 6.

**Figure 7:**
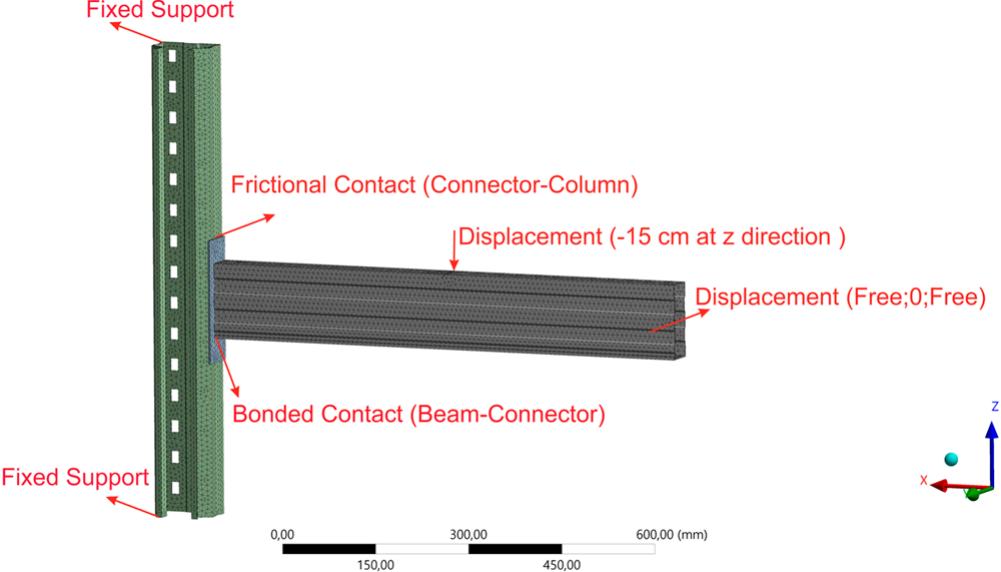
Boundary conditions for the developed numerical model.

Based on the validation study results, it was decided to use the 8 mm element size in the analyses together with the determined boundary conditions shown in Figure 7. The number of elements and nodes are 58040 and 118425, respectively.

#### 3.3.2 Moment–Rotation (*M*–θ) Behavior and Stiffness Results

The moment (*M*) was calculated using the equation *M* = *P* × *d* in the nodal region, which is the intersection of the column and beam axes. Here, *P* is the applied load and *d* is the real horizontal distance between the connector and the load point. The rotation values occurring at the beam end were calculated as presented in the experimental procedure.

**Figure 8:**
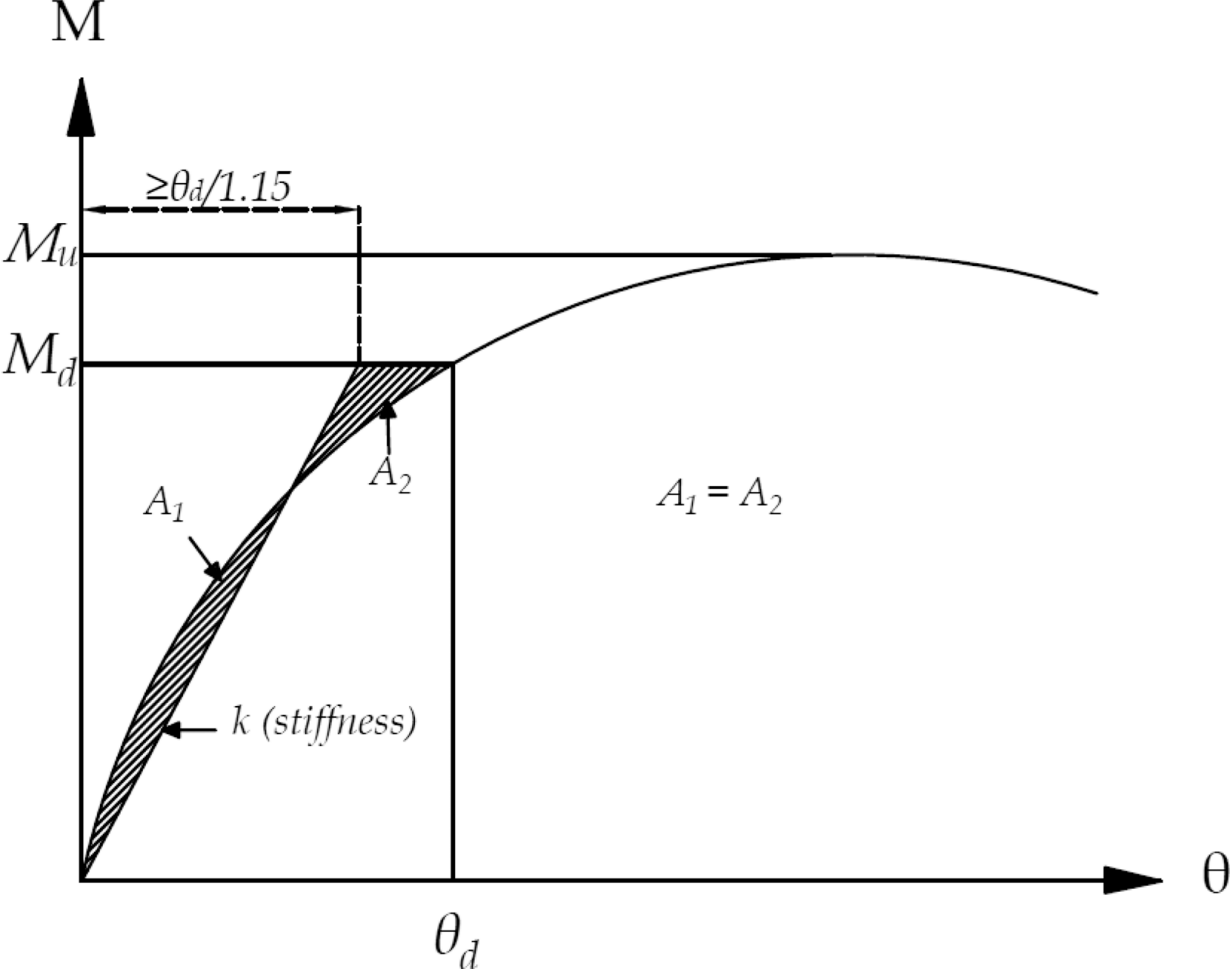
Derivation of the joint’s rotational stiffness.

In the moment–rotation curves, the initial part that corresponds to the rotational stiffness (*k*_0_) exhibited linear behavior as expected. Before the maximum moment is attained, the moment–rotation curve behaves nonlinearly after the linear portion. This rotation in the joint indicates that the joints can be categorized as semirigid joints.^3^ The rotational stiffness value of the joint was calculated using the equal area method shown in Figure 8. Initial linear behavior in the moment–rotation curve depends on good fabrication and design of the column and connector.^33,34^

#### 3.3.3 Effect of Temperature on Connection Performance

The joint behavior information on the tested beam-to-column connections (ultimate moment capacity, rotation, and initial stiffness values) according to the different protection zones are given in Table 3. In this section, the protection cases of beam (B), upright (U), and connector (C) are compared with the case of all protected and not exposed to fire (original at 23 °C).

**Table 3:** Numerical Results in Terms of Ultimate Moment, Rotation, and Initial Stiffness

				performance increment	
specimen ID	ultimate moment (*M*_u_) (kN·m)	rotation (rad)	initial stiffness (*k*_0_) (kN·m/rad)	(±*M*_u_%)	(±*k*_0_%)
original at 23 °C	5.24	0.36	74.78	11.02	2.76
all-unprotected	4.72	0.36	72.77	0.00	0.00
all-protected	5.06	0.36	78.05	7.20	7.26
protected-(B)	4.98	0.36	69.48	5.51	–4.52
protected-(U)	4.77	0.36	73.55	1.06	1.07
protected-(C)	4.75	0.36	74.48	0.64	2.35
protected-(B+C)	5.01	0.36	71.47	6.14	–1.79
protected-(B+U)	5.03	0.36	71.49	6.57	–1.76
protected-(U+C)	4.80	0.36	75.38	1.69	3.59

It was observed that rotation values (θ) of the test specimens did not exhibited any measurable change based on the protection technique. This consequence depends on the presence of low rotation values owing to the ordinary behavior of the joint. However, ultimate moment capacity (*M*_u_) and initial stiffness (*k*_0_) values yield significant inferences for the purpose of exposing the efficiency of fire protection technique on the rack system components.

Initially, it was seen that fire effect reduced the ultimate moment capacity more than 11% and the initial stiffness nearly 3% considering original and all-unprotected specimens. When the all-protected and all-unprotected specimens are compared, ultimate moment capacity and initial stiffness values increase more than 7%, which represents a remarkable structural performance contribution of the paint protection. Relatively low levels of performance increment are achieved with the individual protection of upright (U) and connector (C) and combined protection of these two members (U+C).

Nevertheless, protection of beam (B) in the individual or binary combinations demonstrates divergent outcomes. This divergence is the increase of ultimate moment capacity alongside the decrease of initial stiffness. From the viewpoint of structural engineering discipline, strong beam–weak column is not a desirable situation considering the integrated behavior of a structure. Even if it does not accurately reflect the current situation, protection of beam and allowing degradation of other members result in excessive stress concentration at the joint. High level of deformation or even fraction is the subsequent results that occur at the joint zone, especially the connector tabs. Increment of stiffness gap between beam and upright–column couple causes this outcome.

**Figure 9:**
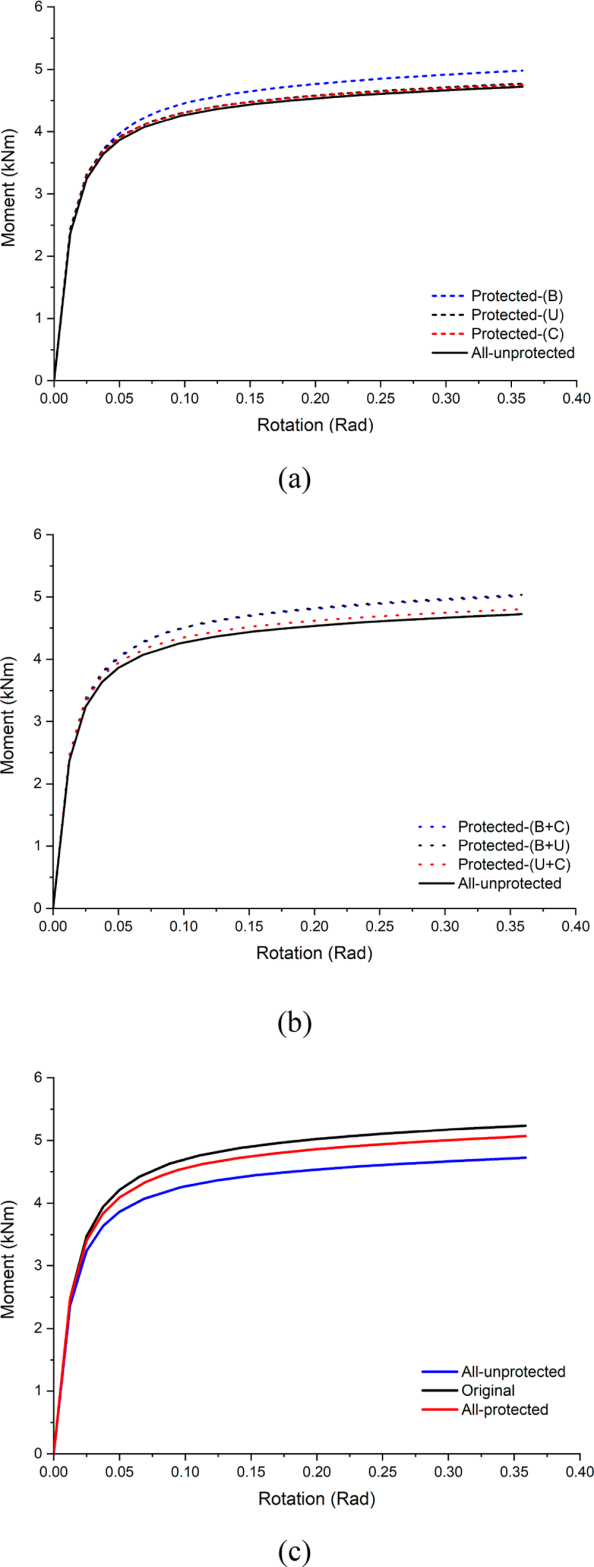
Moment–rotation results of the specimens: (a) individually protected with all-unprotected; (b) binary protected with all-unprotected; (c) all protected and unprotected with original.

In brief, it is possible to state that applying intumescent coating on the total rack system for fire protection increases the fire performance of the connection in a significant manner, in terms of ultimate moment capacity and rotational stiffness. Figure 9 presents the moment–rotation results based on the fire protection zones (individually, binary, all-protected, and all-unprotected conditions).

#### 3.3.4 Critical Stress Distribution

**Figure 10:**
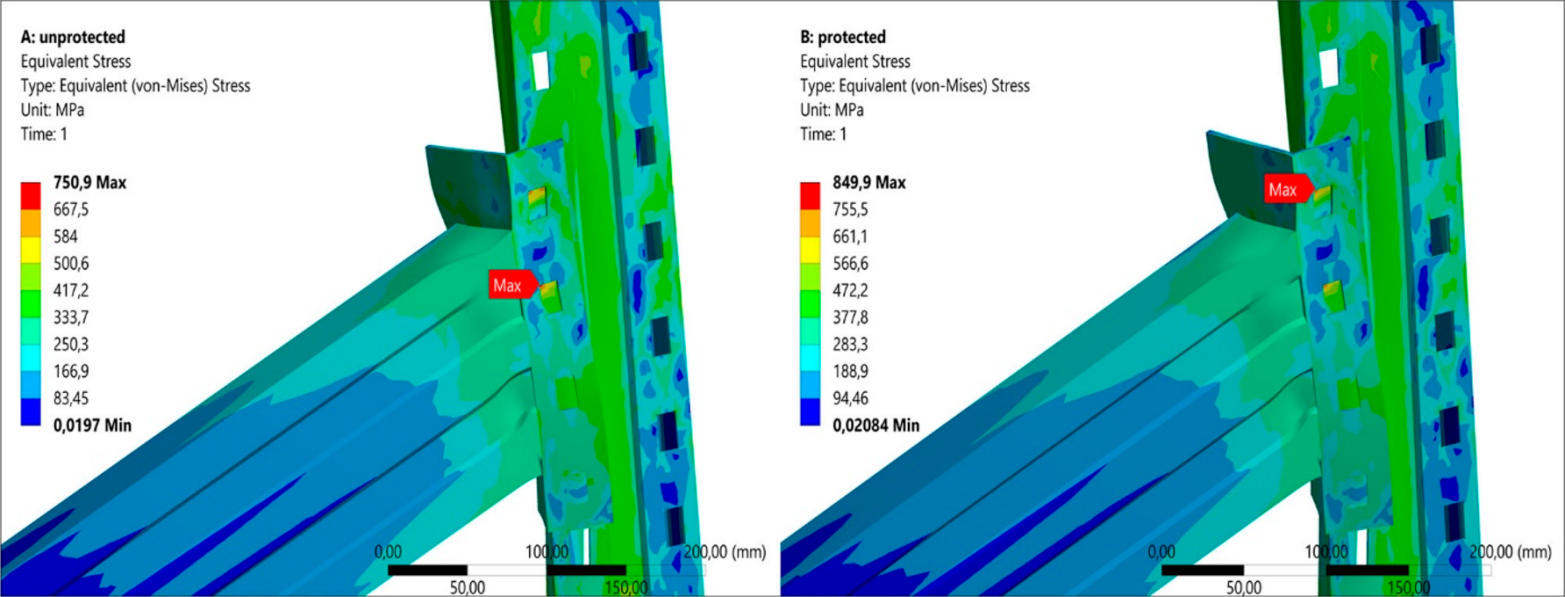
Equivalent (von-Mises) stresses due to protection zones: (a) all-unprotected; (b) all-protected.

Numerical analysis results are demonstrated in Figure 10. Additionally, relative maximum and average von-Mises stress percentages at the ultimate moment levels are provided in Table 4. These relative values have been calculated by equalizing ultimate moment capacities of all the specimens in order to perform a reliable comparison.

**Table 4:** Relative von Mises Stress Levels for the Test Specimens

specimen ID	ultimate moment (*M*_u_) (kN·m)	σ_max_^vM^ (MPa)	σ_avg_^vM^ (MPa)	Δσ_max_^vM-total^ (±%)	Δσ_avg_^vM-total^ (±%)
original at 23 °C	5.24	827.40	139.75	–0.75	–1.54
all-unprotected	4.72	750.90	127.86	0.00	0.00
all-protected	5.06	849.90	135.77	5.58	–0.94
protected-(B)	4.98	809.40	132.43	2.16	–1.83
protected-(U)	4.77	787.10	130.53	3.72	1.02
protected-(C)	4.75	743.20	128.37	–1.65	–0.23
protected-(B+C)	5.01	800.30	132.97	0.41	–2.02
protected-(B+U)	5.03	849.70	135.24	6.18	–0.74
protected-(U+C)	4.80	791.10	131.07	3.60	0.81

These results reveal that the most critical stress level is observed around the connector tabs. Maximum stress level of the all-unprotected specimens is less than that of all-protected specimens, which could be assumed as unexpected at first sight. However, stress distribution throughout the whole specimens should be considered for comprehending the precise effects of fire and fire protection with intumescent coating. In order to explain this divergence, it should be noted that lateral surfaces of the tabs are the most stressed regions due to their thinner cross sections and the point contact with the tab holes of the upright. Owing to higher initial strength and brittleness of the upright material, stiffness and hardness of the tab holes are exposed to more degradation. Hence, local stresses at the tabs ordinarily decrease for the all-unprotected specimens. Relative stress percentages for the specimens support this statement.

Regarding other protection conditions, individual or binary protection of the connector indicates less relative stress levels than other members (beam or upright).

## 4 Conclusions

This study explores the behavior of beam-to-column connections in storage rack systems exposed to high temperatures using finite element approach. The goal of this study was to derive and commentate moment–rotation (*M*–θ) curves after painting various structural members with varying configurations in order to evaluate the performance of intumescent-coated storage rack systems. The results including stiffness of beam-to-column joints have been obtained by comparing the protection of beams, uprights, and connectors in individual, binary, or all-protected conditions with that in all-unprotected condition against fire effects.

A nonlinear three-dimensional finite element (FE) model was constructed to simulate high temperature experimental studies. Elastic modulus and yield strength values of the steel based on the obtained final temperatures have been determined from the study of Chen et al.^25^ These mechanical properties have been used in the static structural analyses to predict the behavior of the joint producing the moment–rotation (*M*–θ) curves and equivalent (von Mises) stresses. The main outcomes of the study are

- Predicted temperature results of structural steel members (uprights, beams and connector) protected with 1 mm intumescent coating thickness were validated with Eurocode data with a respectable agreement.
- Fire effect reduces ultimate moment capacity more than 11% and initial stiffness nearly 3% considering original and all-unprotected specimens.
- Comparing fully protected and unprotected conditions, the joint’s ultimate moment capacity and initial stiffness values increase more than 7%. This outcome in fire protection technique (applying intumescent coating) certainly improves the connection’s fire performance.
- Protection of beam (B) in the individual or binary combinations leads to the increase of ultimate moment capacity alongside the decrease of initial stiffness. This situation results in excessive stress concentration at the joint, especially the connector tabs.
- The most critical stress level is achieved around the connector tabs, because lateral surfaces of the tabs are the most stressed regions due to their thinner cross sections and the point contact with the tab holes of the upright.
- While protection of the all-rack system members yields significant performance increment in terms of the whole structural behavior, protection of connector in individual or binary conditions ensures acceptable advantages.
